# Isolation and characterization of farm pig adipose tissue-derived mesenchymal stromal/stem cells

**DOI:** 10.1590/1414-431X2022e12343

**Published:** 2022-12-02

**Authors:** G.A. Garcia, R.G. Oliveira, R. Dariolli, M.V.C. Rudge, A.M.P. Barbosa, J.F. Floriano, J.T. Ribeiro-Paes

**Affiliations:** 1 Departamento de Biotecnologia, Faculdade de Ciências e Letras, Universidade Estadual Paulista, Assis, SP, Brasil; 2 Instituto de Ciências Biomédicas, Universidade de São Paulo, São Paulo, SP, Brasil; 3 Laboratório de Genética e Cardiologia Molecular, Instituto do Coração, São Paulo, SP, Brasil; 4 Department of Pharmacological Sciences, Icahn School of Medicine at Mount Sinai, New York, NY, USA; 5 Departamento de Ginecologia e Obstetrícia, Faculdade de Medicina, Universidade Estadual Paulista, Botucatu, SP, Brasil; 6 Departamento de Fisioterapia e Terapia Ocupacional, Faculdade de Filosofia e Ciências, Universidade Estadual Paulista, Marília, SP, Brasil

**Keywords:** Collagenase, Protocol, Stem Cells, Porcine, Adipose tissue

## Abstract

Adipose tissue-derived mesenchymal stromal/stem cells (ASCs) are considered important tools in regenerative medicine and are being tested in several clinical studies. Porcine models are frequently used to obtain adipose tissue, due to the abundance of material and because they have immunological and physiological similarities with humans. However, it is essential to understand the effects and safe application of ASCs from pigs (pASCs) as an alternative therapy for diseases. Although minipigs are easy-to-handle animals that require less food and space, acquiring and maintaining them in a bioterium can be costly. Thus, we present a protocol for the isolation and proliferation of ASCs isolated from adipose tissue of farm pigs. Adipose tissue samples were extracted from the abdominal region of the animals. Because the pigs were not raised in a controlled environment, such as a bioterium, it was necessary to carry out rigorous procedures for disinfection. After this procedure, cells were isolated by mechanical dissociation and enzymatic digestion. A proliferation curve was performed and used to calculate the doubling time of the population. The characterization of pASCs was performed by immunophenotyping and cell differentiation in osteogenic and adipogenic lineages. The described method was efficient for the isolation and cultivation of pASCs, maintaining cellular attributes, such as surface antigens and multipotential differentiation during *in vitro* proliferation. This protocol presents the isolation and cultivation of ASCs from farm pig as an alternative for the isolation and cultivation of ASCs from minipigs, which require strictly controlled maintenance conditions and a more expensive process.

## Introduction

Mesenchymal stromal/stem cells (MSC) have been considered as a promising therapeutic alternative in several preclinical and clinical studies. This is due to their non-immunogenicity, which guarantees in theory the possibility of using these cells in allogeneic transplants, and their therapeutic potential in regenerative and translational medicine due to their cell differentiation potential and immunomodulatory properties (1-10). Adipose tissue is an interesting source for MSC isolation, since biological samples can be acquired in large quantities by less invasive procedures compared to other tissues. Also, it provides a great number of cells that can be cultivated and proliferated *in vitro* (11-18).

Porcine models are frequently used to obtain adipose tissue because they have an abundance of fat. They have been used in several studies in the hepatic field (19), metabolic syndromes (20), and also to understand the repair potential of their extracellular vesicles (21). Pigs are the closest non-primate species to man and share immunological and physiological similarities (22). Isolation and characterization studies of porcine adipose tissue-derived mesenchymal stromal/stem cells (pASCs) are essential for a better understanding of their effect and safe application in regenerative medicine (3,23).

Minipigs or miniature pigs, compared to conventional farm pigs, are easier to handle, spend less on feed and require less space. However, the conditions of the facilities (flooring design, optimal temperature, humidity, and lightning) must be strictly controlled to minimize stress, since the stable physiological state of the animal is fundamental for research success (24). Minipigs are social animals, so they can be housed in small groups. Even in isolation, pigs must have visual, olfactory, and auditory contact with each other to avoid social deprivation (25). Currently, there are no studies about the costs of building these facilities. In addition, considering that minipigs are genetically modified animals, their acquisition tends to cost more. The University of Missouri (26), for instance, commercializes a species of pig for the price of US$2,250/animal (28 days old).

The farm pig is a low-cost alternative source of adipose tissue for experimental studies, since there are no expenses for facility construction, acquisition of the animals, or their maintenance in a bioterium environment. Another advantage of using MSCs isolated from adipose tissue of farm pigs is the constant availability of the material, since these animals are widely bred for human meat consumption. In this context, this study aimed to present an efficient, feasible, and reproducible methodology for isolation and culture of pASCs.

## Material and Methods

### Statement of animal welfare

The present study was carried out using samples of adipose tissue from farm pigs raised for human meat consumption. The slaughterhouse Assiscarnes - Distribuidora de Carnes Ltda. (Fribom, Brazil) has the SISP seal (Inspection Service of São Paulo State, Brazil; no. 458), granted by the Coordination of Agricultural Defense (Government of São Paulo State, Brazil), which guarantees that the establishment complies with all safety and animal welfare standards.

### Sample collection

**Figure 1:**
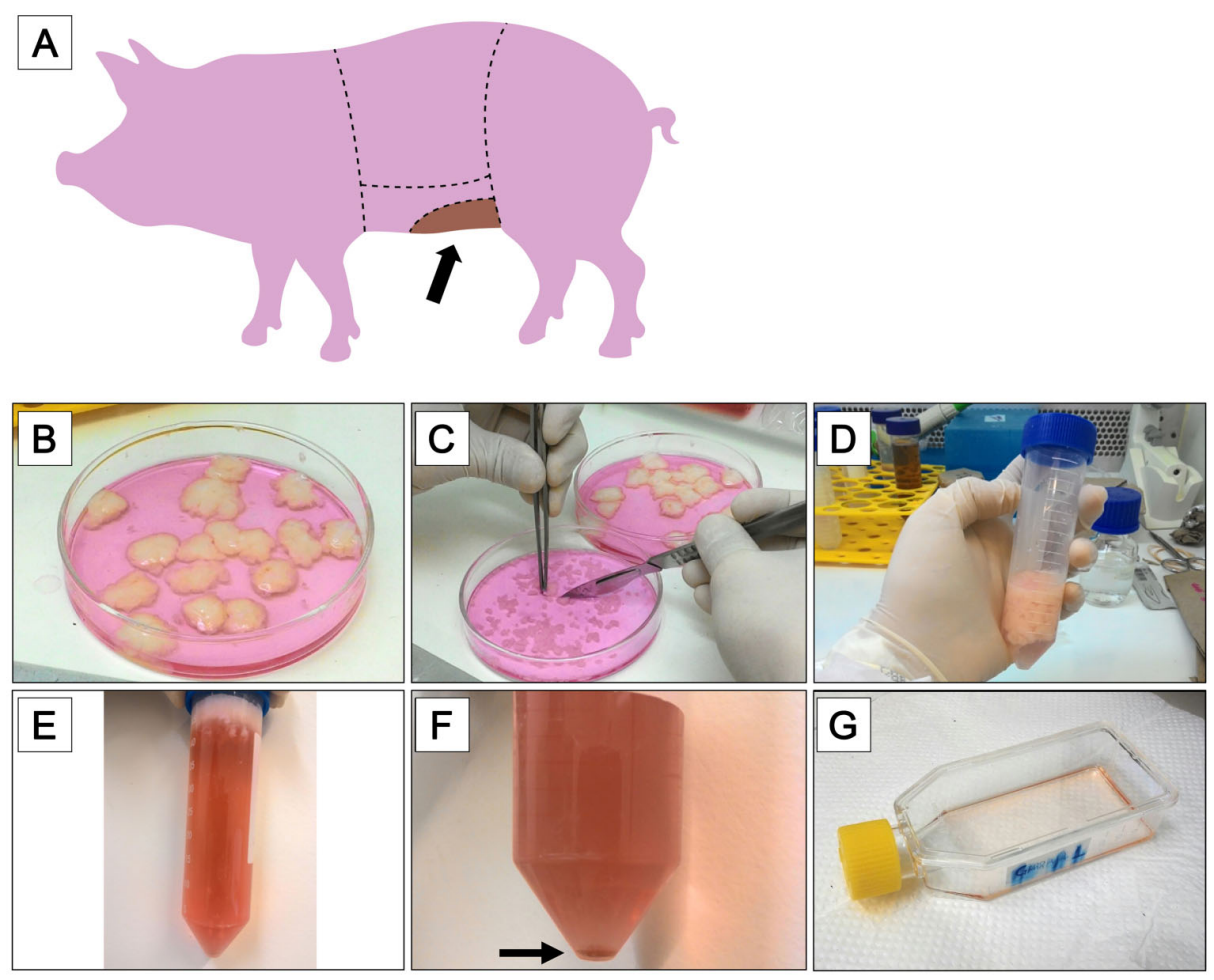
Sample collection and isolation of farm pig adipose tissue-derived mesenchymal stromal/stem cells (pASCs) scheme. **A**, Schematic representation of the farm pig's abdominal region. The darker area indicates the region where the adipose tissue was collected. **B**-**G**, Isolation of pASCs. **B**, Tissue disinfection and fragmentation; **C**, mechanical dissociation; **D**, enzymatic digestion with collagenase; **E**, centrifugation and separation of cells and cell debris by density difference; **F**, second centrifugation and formation of a cellular pellet (black arrow); **G**, cultivation of pASCs in culture flasks.

The material was donated by a commercial slaughterhouse that processes meat for human consumption. One hundred grams of adipose tissue was collected from each animal (n=5; Figure 1A). The material was maintained in phosphate-buffered saline 1X (PBS; LGC Biotecnologia, Brazil) supplemented with 2% antibiotic-antimycotic (AA: 10,000 units/mL of penicillin, 10,000 µg/mL of streptomycin, and 25 µg/mL of Gibco Amphotericin B; Gibco, Thermo Fisher Scientific, USA) for 30 min. Residual skin was removed, and the adipose tissue was fragmented in samples of 25 g each. Each sample was washed 3 times with PBS containing 2% AA 100X for 10 min.

### Preparation of growth medium

The growth medium (GM) was prepared using alpha-MEM culture medium (LGC Biotecnologia) with filtered, sterile, and inactivated 10% fetal bovine serum (FBS; LGC Biotecnologia) and 2% AA (Gibco).

### Isolation of pASCs

The samples were placed in a petri dish containing alpha-MEM culture medium supplemented with 2% AA (Gibco) (Figure 1B). The tissue was cut into small pieces using tweezers and scalpel blades (Figure 1C). The material was transferred to a 50-mL conical and sterile screw cap tube (SARSTEDT, Germany) and the alpha-MEM culture medium was discarded. Collagenase Type I, 125 units/mg (Gibco, Thermo Fisher Scientific) was added at a concentration of 0.15% for enzymatic digestion (30 mL for each 15 g of adipose tissue) at 37°C for 1 h, under agitation (Figure 1D). If a shaking water bath is not available, it is possible to manually agitate the tubes every 5 min, ensuring the detachment of cells. FBS was added to neutralize the enzyme (1:1). The tubes were centrifuged for 10 min at 390 *g* and 20°C. Supernatant adipocytes was removed (Figure 1E) and centrifugation was performed once again. Supernatant adipocytes were removed and the enzyme was neutralized with FBS. The formed cell pellet (Figure 1F) was resuspended in 5 mL of alpha-MEM culture medium. The material was filtered through a 70-μm Falcon cell strainer (Fisher Scientific, Thermo Fisher Scientific), and an aliquot was collected for cell counting in a Neubauer Chamber (Kasvi, Brazil) as described by Freshney (15). Cells (2×10^4^) were plated onto a T-25 tissue culture flask (BD Biosciences, USA) and filled with GM (Figure 1G). The culture flasks with cells were incubated in a 5% CO_2_ incubator at 37°C and after 2 h of plating, and the GM was then discarded. Discarded GM can be seeded in new culture flasks, increasing the number of cells in cultivation. The flasks containing the adherent pASCs were washed with PBS for elimination of cell debris and mature adipocytes, which is crucial to improve cell adhesion and proliferation. Fresh GM was added and cells were cultivated in a 5% CO_2_ incubator at 37°C.

### Maintenance and cell proliferation

During the entire culture process, half of the cell growth medium was discarded every 48 h and fresh GM was added at the same proportion. Upon reaching 85-90% confluence, the cells were dissociated and replicated (passaged) in new flasks (T-75 and T-175, consecutively) for proliferation. The culture was replicated in a new culture flask by discarding the alpha-MEM culture medium, and cells were washed three times with 4-10 mL of PBS (depending on T-flask volume capacity). Enough trypsin-EDTA (0.25%) with phenol red (Gibco, Thermo Fisher Scientific) solution was added to cover the bottom of the flask and cells were incubated for a maximum of 7 min at 37°C to detach the cells from the surface of the flasks. Trypsin (Gibco) was neutralized by adding alpha-MEM culture medium in the same proportion. The cellular solution was transferred to a conical tube (SARSTEDT) and centrifuged for 10 min at 390 *g* and 20°C. The supernatant was discarded and the cell pellet was resuspended in 1 mL of culture medium. The cells were plated in a new culture flask at 1×10^5^ cells/cm^2^. Enough GM was added to cover the bottom of the flask.

### Proliferation curve

In order to evaluate cellular proliferation *in vitro*, the following analysis was performed using pASCs at passage 3. Cells were harvested using trypsin as described in “Maintenance and cell proliferation”. A total of 30 wells with 1×10^3^ cells each was seeded onto 12-well plates. One milliliter of GM was added in each well. The plates were maintained in a CO_2_ incubator with 5% O_2_ for 10 consecutive days at 37°C. Cells were harvested from 3 wells, and a cell count was performed every 24 h. GM was replaced daily in all wells. The average number of cells obtained each day was calculated and the data was plotted in GraphPad Prism software, version 7.0 (GraphPad Software, USA) to generate the proliferation curve. Samples were placed in a petri dish containing alpha-MEM culture medium supplemented with 2% AA.

### Population doubling time (PDT)

Complementing the analysis of cellular proliferation, the cell population doubling capacity was investigated considering the log phase of the proliferation curve as described by Freshney (15) using the formula PDT = [Ln(2) × t] / [Ln (N2 / N1)], where N2 is the final cell concentration, N1 is the initial cell concentration, and t is the time interval from N1 to N2.

### *In vitro* differentiation of pASCs

To evaluate pASCs differentiation, cells were cultivated to passage 3 and maintained for 7 and 21 days, respectively, in adipogenic and osteogenic differentiation kit solutions (Gibco, Thermo Fisher Scientific) following the manufacturer's instructions. Adipogenic differentiation was confirmed for positive staining by Oil Red O (Sigma-Aldrich, USA). The medium was removed after 7 days, and the wells were washed with PBS. Cells were fixed with 1% formalin for 15 min. Wells were washed again with 60% isopropanol and Oil Red O (Sigma-Aldrich) was added for 10 min. The wells were washed and visualized under a microscope to detect lipid vacuoles. For osteogenic differentiation, the medium was removed after 21 days, and the wells were washed with PBS. Cells were fixed with 4% formaldehyde for 30 min and washed twice with distilled water. Alizarin Red S 2% (pH 4.2) (Sigma-Aldrich) was added and maintained for 3 min for staining. Then, the wells were again washed with distilled water and taken for microscope viewing.

### Flow cytometry assay

The pASCs immunophenotype analysis was performed with cells at passage 3 by flow cytometry in a FACSCalibur flow cytometer (USA). The choice of antibodies was based on the recommendations of the International Society of Cellular Therapy (ISCT) and the International Federation for Adipose Therapeutics and Science (IFATS) (13,14), and their use has been reported in several studies for the characterization of this cell type (3,27-34). Cells were incubated, separately, with purified antibodies CD29 (1:2000, 552369, BD Biosciences) and CD90 (1:2000, 555593, BD Biosciences), and primary antibodies CD45 (340943, BD Biosciences) and CD31 (555027, BD Biosciences) for 30 min at 4°C. Samples with purified antibodies were further incubated with secondary antibody Alexa Fluor 647 (Invitrogen, Thermo Fisher Scientific, A-21235) for 30 min at 4°C. Table 1 describes each sample of this protocol. A total of 10,000 events were acquired in a flow cytometer. Cell Quest software (BD Biosciences) was used for data analysis.

**Table 1:** Sample identification used for flow cytometry assay of farm pig adipose tissue-derived mesenchymal stromal/stem cells.

	Sample identification				
	1	2	3	4	5
Primary antibodies (µL)	-	CD31 (-) 2	CD29 (+) 2	CD90 (+) 5	CD45 (-) 5
Secondary antibody Alexa Fluor 647 (µL)	-	-	0.5	0.5	-
PBS (µL)	100	98	97.5	94.5	95
Total volume (µL)	100	100	100	100	100

Components and volumes used for each sample tested by flow cytometry assay. PBS: phosphate-buffered saline.

## Results

This protocol presents a method for the isolation and culture of pASCs collected after pigs' slaughter. These cells are an important tool for experimental studies, since they can be isolated more easily and in large quantities, from animals that do not need a bioterium environment for maintenance.

**Figure 2:**
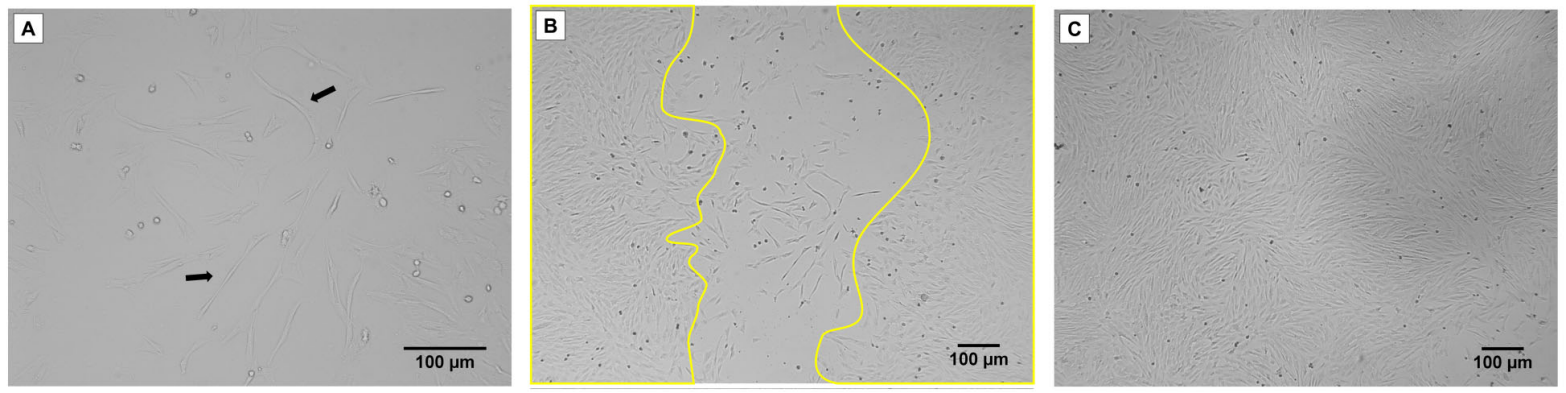
Farm pig adipose tissue-derived mesenchymal stromal/stem cells (pASCs) morphological characteristics in culture. **A**, Fibroblastic aspect and hyaline cytoplasm of pASCs in a proliferation process; **B**, colony formation; and **C**, culture cell confluence (85-90%) after seven days of plating. Scale bar 100 μm.

The process of mechanical cell dissociation followed by the enzymatic digestion for pASCs isolation provided a total of approximately 8×10^4^ cells/mL, considering the final concentration, equally divided and plated onto four T-25 culture flasks. Initial pASCs attachment in the flasks' surface started 2 h after plating. Colony-forming ability was observed from day 2 (Figure 2B, yellow rim) and cellular confluence (85-90%) was reached in 5 days of culturing (Figure 2C). Subsequently, pASCs were replicated in larger culture flasks and cultivated until passage 3, when cells were used to perform the proliferation and characterization tests.

**Figure 3:**
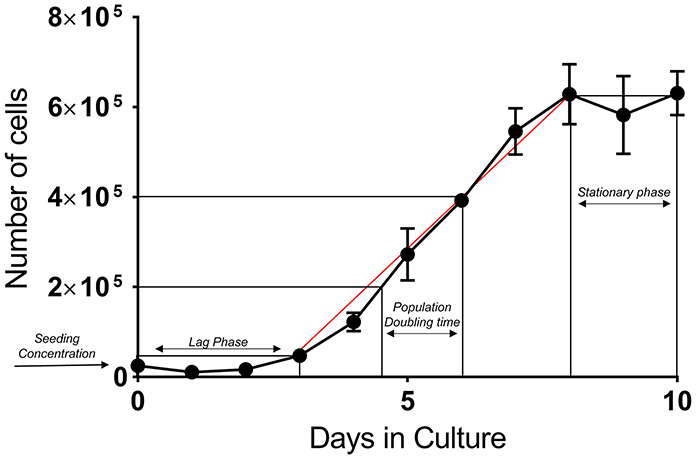
Proliferation curve of the farm pig adipose tissue-derived mesenchymal stromal/stem cells (pASCs) in 10 days in culture. Different phases are indicated in the plot, including exponential phase (red line). Population doubling time (PDT) was derived from the middle region of this best-fit red line (n=3). Data are reported as means and SE.

The proliferation potential of pASCs *in vitro* was evaluated for 10 consecutive days and the data were used to generate the proliferation curve (Figure 3). The first days after plating is the period in which cells adapt and adhere to the surface (lag phase) before starting the self-division process. After this initial phase, cells exhibited exponential proliferation (log phase) between days 3 and 8, which was stabilized in the following days (stationary phase). There was no decline in the number of cells (cell death) at the end of the tenth day. Population doubling time (PDT) was calculated in triplicate based on the proliferative curve for pASCs at passage 3, considering days 4 to 7, and a value of 33.86±5.52 h was obtained (mean and standard deviation of the mean).

**Figure 4:**
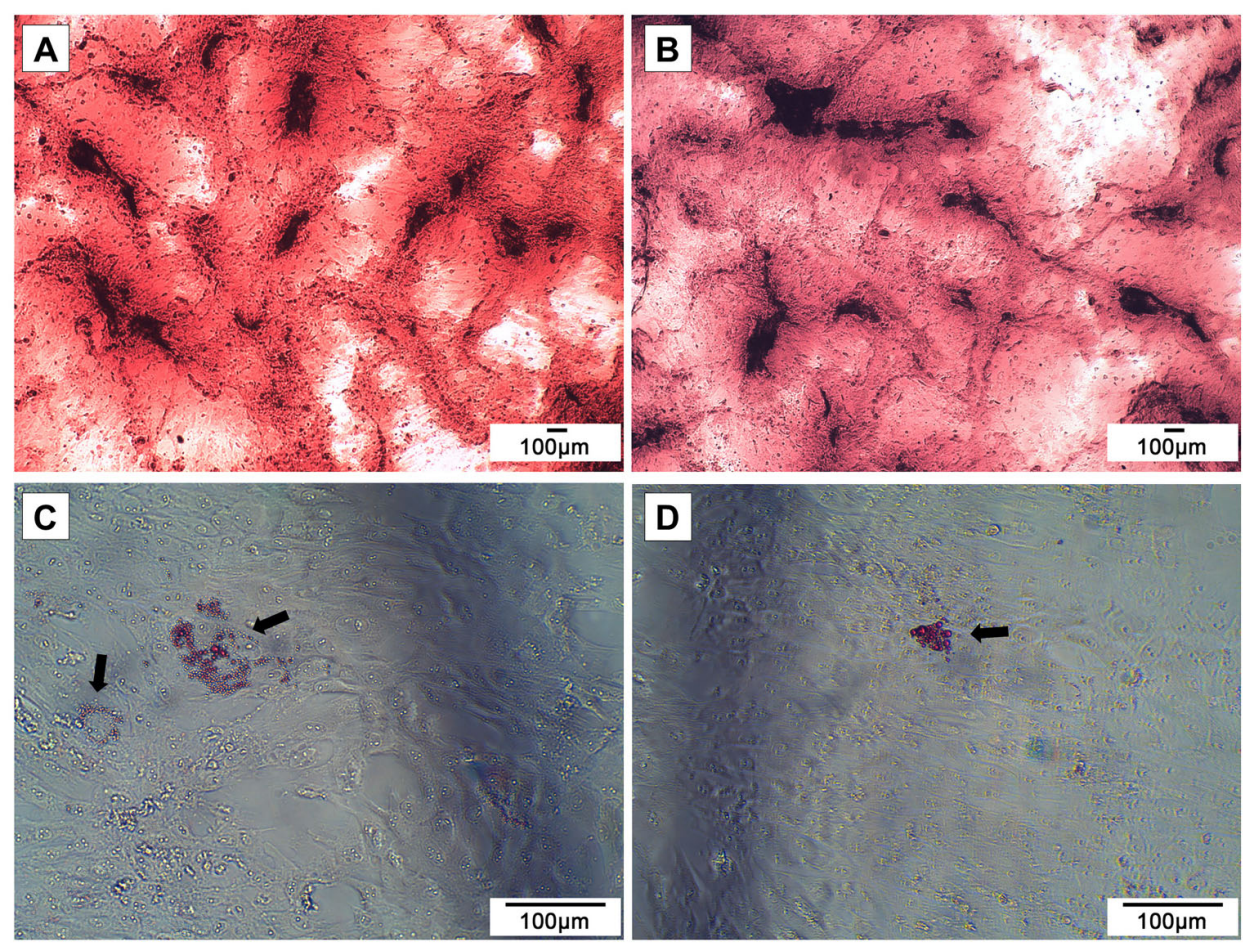
Farm pig adipose tissue-derived mesenchymal stromal/stem cells (pASCs) characterization. Adipogenic (**A** and **B**) and osteogenic (**C** and **D**) pASCs differentiations at passage 3 after Oil Red O and Alizarin Red S staining, respectively. Black arrows indicate stained adipose vacuoles. Scale bar 100 μm.

The *in vitro* differentiation potential was evaluated in order to characterize pASCs as mesenchymal cells. Cells at passage 3 were cultivated with specific induction media and then analyzed with Alizarin Red S and Oil Red O staining. The osteogenesis process was observed by the presence of calcium deposits covering the cells (Figure 4C and D), while the adipogenic differentiation resulted in the formation of intracellular lipid vacuoles (Figure 4A and B).

Farm pASCs were also characterized by immunophenotyping analysis. Cultured pASCs at passage 3 expressed specific mesenchymal surface markers CD29 and CD90 (98.9 and 95.1%, respectively), while being negative for endothelial marker CD31 and leucocyte marker CD45 (0.3 and 0.1%, respectively) (Supplementary Figure S1).

## Discussion

ASCs have been tested as a therapeutic alternative in several studies. However, it is crucial to deepen the knowledge about this cell type, evaluate its therapeutic potential, and make its use in transplants safer. This study presents a protocol for the isolation and proliferation of pASCs based on mechanical dissociation and enzymatic digestion methods.

Concomitant with the initial attachment, pASCs morphology changed after seeding to a similar fibroblast phenotype typical of mesenchymal cells (14). pASCs presented evident hyaline cytoplasm and elongated cell extension. These same characteristics were observed in similar cells derived from domestic pigs raised in a bioterium, as described by Dariolli et al. (3).

pASCs isolated from farm pigs were able to double their number of cells, by *in vitro* proliferation, every 33 h. Schweizer et al. (33) evaluated PDT for swine MSCs isolated from omental and subcutaneous fat and observed longer times for cells at passage 1 (55.1±14.71 h and 50.36±16.53 h, respectively), while the same cells at passages 2 to 5 presented PDT between 20-35 h. Compared with domestic pASCs (3), farm pASCs presented faster PDT, *in vitro*. However, those protocols were applied for cells at different passages and performed over different times.

The *in vitro* differentiation potential was evaluated in order to characterize pASCs as mesenchymal cells (13,14). The cells differentiated in both adipogenic and osteogenic lineages, confirmed by the formation of adipose vacuoles inside the cellular cytoplasm and the presence of calcium deposits, respectively.

According to IFATS and ISCT, ASCs immunophenotyping should include at least two negative and two positive markers in the same analysis and should be performed using primary stable positive antibodies: CD13, CD29, CD44, CD73, CD90, and CD105 (>80%) and primary negative antibodies: CD31, CD45, and CD235a (<2%). The percentage of farm pASCs marked by CD29 and CD90 was higher than 95%, while CD45 and CD31 was lower than 0.5%. These results also characterize the farm pASCs as being cells of the mesenchymal type since they present cell surface antibodies typical of this cell type (3,27-34).

It is important to highlight that there is variation in cell characteristics during *in vitro* culture, such as the potential for differentiation, even when isolated from animals of the same species (35). For the present study, analyses of cell proliferation, PDT, and characterizations were performed with a pool of pASCs isolated from 5 animals. Further studies with this cell type are necessary to investigate the influence of the genetic variability of the animals on the results. In this way, it will be possible to expand the basic knowledge about pASCs and verify the potential application and safety of these cells in therapeutic tests.

This protocol describes an adapted and effective methodology for the isolation of ASCs from farm pigs. Each sample collected provided 8×10^4^ cells, which adhered and proliferated in culture flasks, doubling in number every 2 days, approximately. Moreover, pASCs maintained a mesenchymal morphological aspect throughout the culture period, and they presented immunophenotype and differentiation capacity typical of a mesenchymal cell in characterization tests.

The farm pig adipose tissue is a low-cost alternative source for ASCs isolation, since the biological material is obtained from farm animals, without the costs for animal acquisition and maintenance in a bioterium. In addition, farm pigs are an abundant source of adipose tissue, as these animals are raised on a large and constant scale for human meat consumption. Therefore, this study proposed a method for the isolation and proliferation of ASCs from farm pigs that can be widely applied in tissue engineering and regenerative medicine studies.

## Supplementary Material

Click to view [pdf].
